# Primary and reproductive healthcare access and use among reproductive aged women and female family planning patients in 3 states

**DOI:** 10.1371/journal.pone.0285825

**Published:** 2023-05-24

**Authors:** Liza Fuentes, Ayana Douglas-Hall, Christina E. Geddes, Megan L. Kavanaugh

**Affiliations:** 1 Formerly of the Research Division, Guttmacher Institute, New York, New York, United States of America; 2 Research Division, Guttmacher Institute, New York, New York, United States of America; Tulane University School of Public Health and Tropical Medicine, UNITED STATES

## Abstract

Public funding plays a key role in reducing cost barriers to sexual and reproductive health (SRH) care in the United States. In this analysis, we examine sociodemographic and healthcare seeking profiles of individuals in three states where public funding for health services has recently changed: Arizona, Iowa, and Wisconsin. In addition, we examine associations between individuals’ health insurance status and whether they experienced delays or had trouble in obtaining their preferred contraception. This descriptive study draws on data collected between 2018 to 2021 in two distinct cross-sectional surveys in each state, one among a representative sample of female residents aged 18–44 and the other among a representative sample of female patients ages eighteen and older seeking family planning services at healthcare sites that receive public funding to deliver this care. The majority of reproductive-aged women and female family planning patients across states reported having a personal healthcare provider, had received at least one SRH service in the preceding 12 months, and were using a method of birth control. Between 49–81% across groups reported receiving recent person-centered contraceptive care. At least one-fifth of each group reported wanting healthcare in the past year but not getting it, and between 10–19% reported a delay or trouble getting birth control in the past 12 months. Common reasons for these outcomes involved cost and insurance-related issues, as well as logistical ones. Among all populations except Wisconsin family planning clinic patients, those with no health insurance had greater odds of being delayed or having trouble getting desired birth control in the past 12 months than those with health insurance. These data serve as a baseline to monitor access and use of SRH services in Arizona, Wisconsin, Iowa in the wake of drastic family planning funding shifts that changed the availability and capacity of the family planning service infrastructure across the country. Continuing to monitor these SRH metrics is critical to understand the potential effect of current political shifts.

## Introduction

Public funding, including Medicaid and other government-funded programs that support low- or no-cost family planning services provided by safety net clinics, plays a key role in reducing cost barriers to sexual and reproductive health care in the United States. In 2016, some 9 million women received publicly supported contraceptive care, either through Medicaid or from Title X-funded clinics or other publicly supported clinics [1]. The Title X program is the only federal program in the United States with the mission “to provide individuals the information and means to exercise personal choice in determining the number and spacing of their children” [2]. In the aftermath of the 2016 United States presidential election, significant reduction in support for public funding for sexual and reproductive health (SRH) care was anticipated; thus, we designed the Reproductive Health Impact Study (RHIS) to document and track state- and federal-level policy changes in publicly funding family planning services, and the potential effects on the healthcare facilities, providers and patients who rely on these funding sources. The project focuses on four states with distinct reproductive health policy landscapes—Arizona, Iowa, New Jersey, and Wisconsin—and encompasses multiple research efforts, including surveys and interviews with both healthcare providers and patients [3].

**Fig 1:**
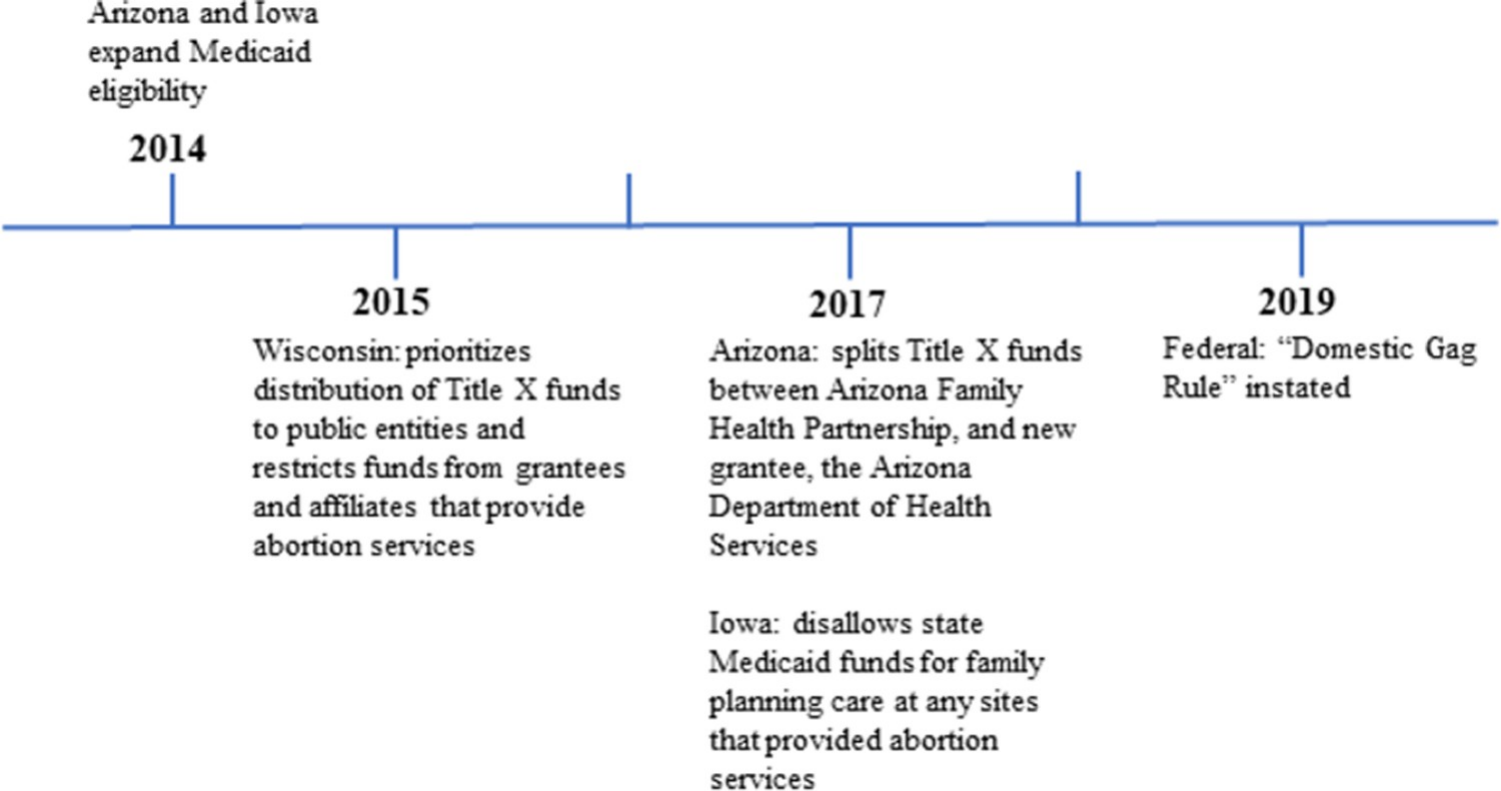
Timeline of family planning and healthcare funding policy implementation in study states.

In this analysis, we focus specifically on individuals in the three states where public funding for sexual and reproductive health services had recently changed when the RHIS commenced: Arizona, Iowa, and Wisconsin (Fig 1). We consider public funding for family planning care as coming from federal, state or local government funding through programs such as Title X, Medicaid or the federally qualified health center program. In 2014, Arizona and Iowa expanded Medicaid eligibility under the Affordable Care Act (ACA), while Wisconsin has not done so. [4]. All Wisconsinites with poverty-level incomes are eligible for Medicaid, and the state, has a state plan amendment (SPA) that extends Medicaid eligibility specifically for family planning services [5, 6]. In 2015, the Wisconsin state legislature passed a law that prioritized distribution of Title X funds to government agencies and restricted those funds from going to organizations that that provide or refer patients for abortion services, such as Planned Parenthood. In 2017 Arizona split Title X funds between the longtime sole Title X grantee and the Arizona Department of Health Services, a new grantee that state law compelled to apply for this grant. Also in 2017, Iowa left the federal Medicaid family planning program and set up its own state program, which explicitly disallowed Medicaid coverage for family planning care at any facilities that provided abortion care or referrals. Emerging evidence suggests that people seeking SRH care after these policy changes experienced disruptions in their ability to obtain services [7]. For example, the number of people enrolled in the Iowa state-funded family planning program dropped by 75% in its first year [3].

In addition to these state-specific changes in public healthcare funding, the 2019 Title X Final Rule, also known as the “Domestic Gag Rule,” further disrupted publicly funded family planning care across the United States [8]. The 2019 Title X Final Rule imposed several significant restrictions on programs receiving Title X funds, including requiring physical and financial separation of abortion services from all other family planning services, barring sites from providing, referring, encouraging, promoting, or advocating for abortion access, and compelling them to refer all pregnant patients for prenatal health care regardless of patient pregnancy desires [9]. The changed regulations resulted in a nearly 50% reduction in the Title X program network capacity; in other words, 1.5 million fewer female family planning patients were seen under Title X in 2020 compared to 2018. Between 2018 and 2020, the number of female contraceptive patients served by sites receiving Title X funds dropped by 60% in Arizona, nearly 50% in Iowa, and 80% in Wisconsin, due to a combination of the Trump Administration’s Final Rule and the COVID-19 pandemic [10].

Substantial research has documented associations between individuals’ demographic characteristics and both general healthcare and SRH service use and outcomes [11, 12]. It is also well-documented that cost is one of the most persistent barriers to accessing healthcare in the United States [13, 14]. Given fluctuations in sources of family planning care funding, and the subsequent shifts in service capacity and delivery within and among clinics reliant on them, understanding the need for, access to, and use of sexual and reproductive health care among people affected by these multiple policy changes during the recent time period is critical.

Therefore, we use cross-sectional survey data from individuals living in states subject to these policy changes soon after their implementation to describe their general and SRH care experiences. We include measures of general healthcare access because the policy contexts in which the groups under study are seeking care may separately and differentially affect general healthcare access and access to SRH care, and we want to make explicit that one is not a proxy for the other. Presenting both domains allows for a richer description of the overall healthcare access context in which people are seeking SRH care. For example, a 2018–2019 survey among Ohio women revealed that more than a quarter of this group was unable to access general healthcare whereas about 10% had experienced delays or difficulties in accessing contraceptive care, suggesting that the ability to obtain contraceptive care is not an indicator of general healthcare access, and vice versa [15].

The two populations examined include: 1) women seeking SRH services at publicly funded health care sites, including Title X sites that are designed to explicitly serve low-income individuals, and 2) representative samples of states’ female reproductive-aged populations. While the first group represents individuals already using publicly funded family planning care in each state, the latter grouping encompasses a broader pool of individuals who may desire SRH care but may have varying potential need for public funding support for that care. Both of these populations may be affected by changes to policies on how family planning care can be delivered within these states, in varying ways. Our objective is to examine sociodemographic and healthcare seeking profiles of these two groups, with a focus on understanding barriers to family planning care within each. These findings are meant to be used both as point estimates for these measures during this period as well as baseline, comparison points for subsequent surveys assessing trends in these metrics. In addition, we examine associations between individuals’ health insurance status and whether they experienced delays or had trouble in obtaining their preferred contraception.

## Methods

### Data

This descriptive study draws on data from two distinct cross-sectional surveys in Arizona, Iowa, and Wisconsin, for a total of six datasets. Each survey was fielded for several months between 2018 and 2021. One survey, the Survey of Women (SoW), was conducted among a representative sample of reproductive-aged (18–44) female residents, and the other (Family Planning Clinic Patient Survey) was conducted among a representative sample of patients ages fifteen and older seeking family planning services at healthcare sites that receive public funding to deliver this care. For all six datasets, the nonpartisan and objective research organization NORC at the University of Chicago (formerly called the National Opinion Research Center) managed electronic data collection. Survey instruments were similar across all six datasets, focusing on access and barriers to sexual to reproductive health care, contraceptive use, pregnancy attitudes, and other sexual and reproductive health topics; items were drawn from previous surveys of patients at family planning sites and SoWs in Maryland, Delaware, Alabama, and South Carolina [16], among others. While most questions in these domains were identical across all six surveys, there were some differences in survey item wording and response categories. All surveys were pretested for item comprehension and survey flow by NORC and the study team.

#### Surveys of Women

We use baseline data from the Arizona, Iowa, and Wisconsin SoWs, longitudinal population-based surveys conducted by NORC between September 2018 and June 2019 in Iowa and November 2019 and August 2020 in Arizona and Wisconsin. SoWs were conducted in these specific states to align with the broader research efforts under the RHIS. The SoWs are self-administered surveys focused on sexual and reproductive health experiences and attitudes and are representative of the population of reproductive-aged adult women (18–44 years) in each state. A full description of the design and implementation of the SoWs is available elsewhere [17]. Briefly, NORC randomly sampled households in each of these states using address-based sampling methods enhanced with an age-targeted list and demographic information from the American Community Survey; these sampling procedures were similar to related surveys in other states [16, 18, 19]. NORC oversampled census tracts with higher proportions of low-income and non-White populations in each state. NORC made a total of six requests for participation. Initial invitations were mailed to potential participants that included information on how to complete the questionnaire online; paper surveys were later mailed to nonrespondent households.

Individuals were eligible to participate if they self-identified as female, were 18 to 44 years old, and resided in a sampled household. Overall response rates, based on the American Association for Public Opinion Research (AAPOR) Response Rate 3 (RR3) with CASRO assumptions (see The American Association for Public Opinion Research. 2015. Standard Definitions: Final Dispositions of Case Codes and Outcome Rates for Surveys. 8th edition. https://www.aapor.org/AAPOR_Main/media/MainSiteFiles/Standard-Definitions2015_8thEd.pdf), were 38% in Iowa, 32% in Arizona, and 38% in Wisconsin; these resulted in fielding sample sizes of 2,384 in Iowa, 2,055 in Arizona, and 2,041 in Wisconsin. We included in our analyses only individuals assigned female at birth who had completed the survey [20], resulting in analytic sample sizes of 2,313 in Iowa, 2,026 in Arizona, and 2,004 in Wisconsin. To account for nonresponse, base sampling, adjustment for unknown eligibility, and household size, , NORC provided sample weights using poststratification methods to represent the demographics of women aged 18 to 44 in each of the three states. NORC also performed hotdeck imputation on missing information for measures of age, race/ethnicity, nativity, educational attainment, employment status, relationship status and income. We used these imputed versions of these measures for these analyses.

#### Family Planning Clinic Patient Surveys

For data representative of females aged 15 and older seeking family planning care (i.e. receiving a method of birth control, birth control counseling, an STI test, STI treatment, a pregnancy test, or an annual gynecological or well-woman exam including a pap smear or HPV vaccination) at healthcare sites receiving public funding to provide this care, we use baseline data from patients recruited while seeking family planning care at sites in Iowa between May 2018 and February 2019, Arizona between April 2019 and February 2020, and Wisconsin between February 2020 and June 2021. These data were collected as a component of the RHIS. Briefly, facilities for patient recruitment were identified based on three eligibility criteria: 1) receiving public funds (e.g., federal, state or local funding through programs such as Title X, Medicaid or the federally qualified health center program) to provide family planning services; 2) serving 100+ patients assigned female at birth in Iowa annually or 200+ patients assigned female at birth in Arizona or Wisconsin annually based on 2015 annual patient caseloads [21]; and 3) actively providing care as of February 1, 2018 in Iowa, as of March 12, 2019 in Arizona, and as of December 18, 2019 in Wisconsin according to the Guttmacher Institute’s national database of sites providing publicly-supported family planning care. Annual patient caseload thresholds were set to maximize recruitment during the fielding periods and to focus recruitment on those sites that provide the majority of publicly funded family planning care in each state. This database is periodically updated and, additionally, Iowa, Arizona, and Wisconsin sites were updated prior to each state-specific data collection start date.

In Iowa, all 45 eligible facilities were recruited into the study and 22 participated; respondents from five of these sites were removed from the sample (*n*  =  17) because their site of care at baseline served fewer than 100 patients annually based on a 2018 census of publicly funded family planning sites in Iowa conducted by the Guttmacher Institute, data which became available after baseline recruitment into the Iowa study was complete. In Arizona, of 96 eligible facilities, we first purposively sampled all Title X funded sites and all rural sites for an initial 30 sites. We then randomly selected the remaining clinics based on size and stratum, accounting for facility type and population density. Of the final eligible clinic sample of 56 sites in Arizona, 17 participated. In Wisconsin, of 60 eligible facilities, 36 participated in the study.

Frontline staff at each facility invited all patients who were ages 15 and older and seeking family planning care to participate in the study during their appointment intake or registration. For select healthcare sites with large patient caseloads, we supplemented staff’s recruitment efforts with dedicated staff from our own organization in Iowa, NORC in Arizona, and a community-based research organization, Ubuntu Research and Evaluation, in Wisconsin. After the first six months of fielding in Iowa, and for the entirety of the fielding periods in Arizona and Wisconsin, respondents ages 18 and older were given the option of entering in their email address to receive a link to complete the survey outside the site. In Wisconsin, due to the COVID-19 pandemic and significant shifts from in-person to telehealth care, we adapted patient recruitment to also allow clinic staff to recruit patients receiving telehealth family planning care into the study by emailing the survey link to patients.

Participating patients self-administered the electronic questionnaire on a tablet handed out by clinic staff or, if completing outside of the clinic setting, via access to a secure web-based portal. Surveys took approximately 25 minutes for Iowa and Wisconsin participants to complete and 23 minutes for Arizona participants. Participating sites were provided an honorarium of $100. Patients who participated in the study were offered the option of entering a facility-specific raffle for a $50 gift card in Iowa, a $10 gift card to Amazon in Arizona, and the option of selecting a $10 gift card from either Amazon or Walmart in Wisconsin. During the final month of fielding in Iowa, research staff traveled to a handful of remaining healthcare sites to support clinic staff in their recruitment of patients into the study. These patients were offered $10 incentives onsite; given the success of this recruitment strategy, we revised our incentives for patients in fielding subsequent surveys in Arizona and Wisconsin. Across eligible participating facilities, the average response rate based on the number of completed surveys and clinic administrators’ counts of eligible patients during the fielding periods was 20% in Iowa, 42% in Arizona, and 33% in Wisconsin. This resulted in sample sizes of 1,388 respondents assigned female at birth in Iowa, 1,932 in Arizona, and 2,754 in Wisconsin. To align with the SoW survey respondents, we narrowed our analysis to individuals ages 18–44, resulting in analytic sample sizes of 1,224 in Iowa, 1,751 in Arizona, and 2,165 in Wisconsin [7].

For each state-specific Family Planning Clinic Patient survey, we implemented a multi‐stage weighting process to adjust our sample to reflect the universe of patients served at publicly funded family planning centers in 2018 (Iowa and Arizona) or in 2020 (Wisconsin). We adapted this weighting process based on the approach described for these surveys available at https://osf.io/zbg4s/.

### Ethical considerations

All study participants initially provided verbal consent to receiving information about the study at baseline. For all web-based surveys, the first screen presented prior to entering the electronic survey included a statement of informed consent. In order to officially proceed to initiate the survey, respondents pressed a submit button. This passive consent is the field standard for most web surveys for Federal and Non-Federal patients [22]. For respondents who completed paper versions of the Surveys of Women, the questionnaire included a consent statement on the second page.

For the Surveys of Women, NORC’s Institutional Review Board approved the data collection protocols. Given the de-identified nature of the survey data shared with the research team, this secondary data analysis was exempt from further review. All study protocols for the Family Planning Clinic Patient Surveys were approved by both our and our data management partner’s Institutional Review Boards (DHHS identifier IRB00002197).

### Statistical analysis

We present the weighted percent distributions of demographic and socioeconomic characteristics, healthcare access measures, and, among those who had trouble obtaining general and SRH care, the reasons why, by state and survey. We do not statistically compare study outcomes by state or survey because inclusion criteria for SoWs and Family Planning Clinic Patients are not mutually exclusive within each state (respondents to the SoWs may be family planning clinic patients, for example) and different sampling and weighting techniques were applied across the surveys.

Demographic characteristics included categorical variables of age, race/ethnicity, nativity, relationship status, and sexuality. Socioeconomic characteristics included categorical variables of education, employment, imputed data on household income as a percent of the federal poverty threshold, number of months without health insurance in the past year, and type of health insurance coverage. For health insurance, respondents could choose more than one type of health insurance, as applicable.

Healthcare access measures included the following dichotomous variables, descriptions of which largely reflect the item language used in the questionnaires: has a personal healthcare provider; wanted healthcare in the last 12 months but didn’t get it; received at least one of six SRH services in the past 12 months; used any method of birth control in the past 3 months; was delayed or had trouble getting desired birth control in the last 12 months; and experienced “person-centered contraceptive care” at their most recent contraceptive visit in the last 12 months. For the latter, we used the Person-Centered Contraceptive Counseling (PCCC) 4-item measure developed by Christine Dehlendorf, et al [23]. Respondents were asked to rate their most recent birth control healthcare provider on four qualities: “Respecting me as a person”; “Letting me say what mattered to me about my birth control method”; “Taking my preferences about my birth control seriously”; and “Giving me enough information to make the best decision about my birth control method.” Respondents rank each item on a 5-point Likert scale from “Poor” to “Excellent.” The PCCC metric is calculated as the percentage of respondents who gave a top score ("Excellent") on all four items [23].

We then present the crude and adjusted association of not having health insurance (yes or no) with having been delayed or having had trouble getting desired birth control in the last 12 months (yes or no) estimated with logistic regression models, for each survey. Adjusted models are estimated using listwise deletion and analytic samples for unadjusted odds ratios are restricted to the fully adjusted models for comparison. In adjusted models we control for demographic characteristics that have been associated with SRH outcomes in previous literature: age, race/ethnicity, relationship status, and sexual orientation. We also control for measures of socioeconomic status, which could confound the association between health insurance and trouble obtaining a preferred contraceptive: education, income as a percent of the federal poverty threshold, and employment status. Because of sparse data, Hawaiian/Pacific Islander and American Indian/Alaska Native (AI/AN) were collapsed into one category, as they each composed 0–3% of responses in each survey (between 1–30 observations for each cell). All analyses were based on weighted data and completed using the svy command in Stata 17.0 to account for the complex sampling designs.

## Results

### Descriptive statistics

Broadly, family planning clinic patients across all three study states included large percentages of young and low-income individuals, in line with whom these programs are designed to serve (Table 1). The largest age group was 18–24 years old among both family planning clinic patients (43–44%) and women aged 18–44 statewide (24–27%). Between 17% and 18% of women in each state were aged 40–44, whereas 4–5% family planning clinic patients were. Hispanic ethnicity varied, from 66% of Arizona family planning clinic patients to 3% of Iowan women of reproductive age. Native Americans made up 2% of the Arizona family planning clinic patients and fewer than 1% among all other study populations. Between 22% (Arizona family planning clinic patients) and 5% (Wisconsin women of reproductive age) were born outside of the United States. Between 43–46% of statewide populations and 11–27% of family planning clinic patients were married. A majority of all study populations attended some college or more and between 5–18% were unemployed.

**Table 1:** Demographic and socioeconomic characteristics among women aged 18–44 in Arizona, Iowa, and Wisconsin; 2018–2020.

	Arizona		Iowa		Wisconsin	
	Family Planning Clinic Patients	Surveys of Women	Family Planning Clinic Patients	Surveys of Women	Family Planning Clinic Patients	Surveys of Women
**Unweighted n**	**1,754**	**2,026**	**1,226**	**2,331**	**2,166**	**2,004**
	Weighted %	Weighted %	Weighted %	Weighted %	Weighted %	Weighted %
***Demographic characteristics***						
**Age**						
18–24	44%	26%	44%	27%	43%	24%
25–29	26%	20%	23%	18%	25%	19%
30–34	15%	19%	17%	19%	16%	19%
35–39	10%	19%	12%	18%	10%	20%
40–44	4%	17%	4%	18%	5%	18%
**Race and ethnicity**						
White non-Hispanic	25%	47%	74%	83%	60%	77%
Black non-Hispanic	5%	4%	7%	3%	17%	8%
Asian non-Hispanic	1%	2%	3%	2%	2%	3%
American Indian or Alaska Native non-Hispanic	1%	2%	0%	0%	1%	0%
Hawaiian or Pacific Islander non-Hispanic	0%	0%	0%	0%	0%	0%
Multiple races non-Hispanic	2%	3%	4%	8%	7%	4%
Hispanic, Latina, or Latinx	66%	42%	12%	3%	13%	7%
**Born outside of the United States**	22%	17%	8%	8%	5%	8%
**Relationship status**						
Married	26%	43%	14%	48%	11%	47%
Living with romantic partner	22%	22%	32%	20%	26%	21%
Not living with romantic partner	34%	16%	54%	13%	37%	16%
No romantic partner	17%	19%	0%	18%	26%	16%
**Sexual orientation**						
Straight	88%	87%	86%	88%	75%	87%
Lesbian/gay	1%	2%	1%	2%	1%	2%
Bisexual, Other	11%	11%	13%	10%	24%	11%
***Socioeconomic characteristics***						
**Educational attainment**						
Less than high school	9%	6%	4%	2%	4%	3%
High school graduate, GED or alternative	34%	18%	25%	14%	31%	15%
Some college or Associate degree	37%	49%	42%	49%	39%	45%
College graduate or more	20%	27%	29%	34%	25%	36%
**Employment^a^**						
Employed for wages	64%	69%	72%	80%	74%	80%
Out of the workforce	18%	26%	11%	9%	10%	17%
Unemployed	18%	5%	17%	11%	17%	3%
**Income as a % of the federal poverty level**						
Less than 100% FPL	29%	17%	23%	14%	17%	14%
100–199% FPL	28%	21%	25%	20%	20%	17%
200–299% FPL	23%	35%	22%	20%	31%	34%
300% FPL or higher	21%	27%	30%	46%	31%	35%
**Health insurance coverage^b^**						
Employer-based	34%	57%	53%	66%	38%	69%
Affordable Care Act (ACA)	7%	19%	7%	23%	10%	18%
Medicaid	35%	26%	29%	22%	59%	19%
Medicare	1%	2%	1%	2%	1%	3%
Tricare / military insurance	2%	4%	1%	2%	0%	2%
Indian Health Services	2%	2%	1%	0%	1%	1%
Other	3%	0%	2%	1%	3%	1%
No insurance	26%	12%	10%	5%	7%	6%
**Number of months in past year without health insurance**						
None	64%	85%	83%	92%	74%	90%
Less than 1 month	1%	1%	1%	1%	1%	1%
1 to 3 months	3%	2%	4%	2%	5%	2%
4 to 6 months	7%	2%	4%	2%	6%	3%
7 to 12 months	25%	10%	9%	3%	14%	5%

Notes: Samples are weighted to reflect females aged 18–44 (Surveys of Women) or female family planning clinic patients (Family Planning Clinic Patient Surveys) within each state; all variables for all datasets have between 0% (age) and 7% (health insurance) of responses missing.

^a^AZ and WI Surveys of Women respondents could select a single employment category. Respondents in all other surveys could select multiple employment categories. To align responses to the employment status question in other surveys, we have assigned respondents to the highest level of employment reported.

^b^Health insurance coverage types are not mutually exclusive; respondents could indicate as many types of coverage as they had at the time of survey completion and thus do not sum to 100%.

The proportion of those without health insurance ranged from 5% (Iowa women of reproductive age) to 26% (Arizona family planning clinic patients). Between 8% (Iowa women of reproductive age) and 36% (Arizona family planning clinic patients) went some amount of time without health insurance in the past year. Alignment between the SoW samples and state-specific samples of comparable age ranges from the American Community Survey (ACS) on key demographic variables of age, nativity, marital status, educational attainment, income, and insurance coverage are presented in the appendix.

The majority of all study populations had a personal health care provider. (Table 2). Between 22% (Iowa women of reproductive age) and 45% (Arizona family planning clinic patients) wanted healthcare in the past year and did not get it. Most individuals in each study population had received at least one SRH service in the preceding 12 months, with the most common service being receipt of a birth control method or prescription.

**Table 2:** Health care access among women aged 18–44 in Arizona, Iowa, and Wisconsin; 2018–2020.

	Arizona		Iowa		Wisconsin	
	Family Planning Clinic Patients	Surveys of Women	Family Planning Clinic Patients	Surveys of Women	Family Planning Clinic Patients	Surveys of Women
**Unweighted n**	**1,754**	**2,026**	**1,226**	**2,331**	**2,166**	**2,004**
	Weighted %	Weighted %	Weighted %	Weighted %	Weighted %	Weighted %
***General Healthcare***						
**Has a personal provider**	57%	72%	72%	87%	63%	87%
**Wanted health care in last 12 months, but didn’t get**	45%	36%	28%	22%	39%	27%
***Sexual and Reproductive Health***						
**Receive 1 or more reproductive health care in last 12 months^a^**	68%	65%	72%	78%	67%	73%
Birth control method or prescription for birth control	48%	35%	56%	42%	49%	37%
Check-up or medical test related to using a birth control method	33%	27%	40%	32%	32%	24%
Counseling or information about birth control	21%	16%	36%	28%	18%	12%
A discussion about whether they want to become pregnant in the next year	12%	12%	22%	22%	9%	14%
A pregnancy test	27%	15%	32%	22%	17%	14%
A general GYN check-up (annual women’s visit)	37%	52%	51%	67%	37%	60%
**Used a method or methods of birth control in the past 3 months**	83%	81%	86%	80%	92%	82%
**Delayed or had trouble getting desired birth control in last 12 months**	19%	14%	18%	10%	12%	10%
**Past receipt of patient-centered contraceptive care^b^**	57%	49%	81%	58%	66%	57%

Notes: Samples are weighted to reflect females aged 18–44 (Surveys of Women) or female family planning clinic patients aged 18–44 (Family Planning Clinic Patient Surveys) within each state; all variables for all datasets have <4% of responses missing except ’Used a method or methods of birth control in the past 3 months’ for which between 4% (WI FPCC Survey) and 15% (AZ Survey of Women) of responses are missing.

^a^Reproductive health care services in the last 12 months are not mutually exclusive; respondents could select multiple types of care, thus they do not not sum to 100%.

^b^Respondents were considered to have received patient-centered care if they reported having received: a birth control methods or prescription for birth control; a check up or medical related to using a birth control method; or counseling or information about birth control in the prior 12 months and they rated this care as excellent on each of the following four domains: respecting the respondent as a person, letting the respondent say what mattered to them about birth control, taking the respondent’s preferences about their birth control seriously, and giving the respondent enough information to make the best decision about their birth control.

The majority of all study populations reported using any method of birth control in the past 3 months (80–92%). Between 10% (Iowan and Wisconsinite women aged 18–44) and 19% (Arizona family planning patients) reported a delay or trouble getting their desired birth control in the last 12 months. Between 49% and 58% of family planning clinic patients gave their family planning provider an “excellent” rating and 57 to 81% of statewide populations of women of reproductive age did.

Of respondents who were asked to report their reasons for not receiving wanted health care in the past 12 months, the commonly reported ones were: inability to afford the care, not having health insurance, health insurance not covering this care, it was too hard to get to (no transportation, facility too far away, didn’t have time to go), and not knowing where to go (Table 3). Almost one quarter (23%) of those who did not receive wanted health care gave more than one reason for why (not shown). The same reasons were dominant for why people were delayed or had trouble getting their desired birth control in the last 12 months. One-third of people who were delayed or had trouble getting their desired birth control gave more than one reason for why (not shown).

**Table 3:** Reported barriers to healthcare and desired contraception in the last 12 months among women aged 18–44 in Arizona, Iowa, and Wisconsin; 2018–2020.

	Reasons for not receiving wanted healthcare in the past 12 months^a^							Reasons delayed or had trouble getting desired birth control in last 12 months^a^					
	Arizona		Iowa		Wisconsin			Arizona		Iowa		Wisconsin	
	Family Planning Clinic Patients	Survey of Women	Family Planning Clinic Patients	Survey of Women	Family Planning Clinic Patients	Survey of Women		Family Planning Clinic Patients	Surveys of Women	Family Planning Clinic Patients	Surveys of Women	Family Planning Clinic Patients	Surveys of Women
**Unweighted n**	**878**	**664**	**387**	**508**	**858**	**475**		**422**	**228**	**260**	**177**	**266**	**173**
	Weighted %	Weighted %	Weighted %	Weighted %	Weighted %	Weighted %		Weighted %	Weighted %	Weighted %	Weighted %	Weighted %	Weighted %
**Health insurance/cost**													
Couldn’t afford it	57%	48%	58%	48%	61%	49%		34%	50%	36%	31%	33%	30%
Didn’t have health insurance	40%	25%	30%	16%	31%	23%		32%	32%	31%	26%	33%	24%
Health insurance didn’t cover it	13%	24%	22%	24%	23%	29%		6%	16%	10%	16%	8%	10%
Insurance co-pays/deductibles were too high	na	na	na	na	na	na		4%	8%	11%	11%	7%	14%
**Logistic difficulties**													
Too hard to get to (no transportation, facility too far away, didn’t have time to go)^b^	27%	37%	26%	32%	26%	37%		15%	12%	6%	6%	15%	12%
Didn’t know where to go for care	26%	19%	15%	9%	22%	11%		11%	15%	na	6%	8%	6%
Couldn’t get an appointment soon enough	13%	16%	17%	11%	18%	15%		na	na	19%	15%	na	na
Couldn’t get to facility when it was open	9%	7%	12%	9%	5%	7%		12%	8%	8%	10%	11%	10%
Method wasn’t available at facility	na	na	na	na	na	na		8%	2%	6%	4%	6%	12%
Couldn’t get through to facility on telephone	na	na	na	na	na	na		na	na	6%	14%	na	na
**Provider/Facility**													
Usual facility is permanently closed	3%	3%	1%	3%	1%	3%		na	1%	na	na	na	2%
Facility no longer offered method	na	na	na	na	na	na		8%	2%	6%	4%	6%	12%
Once arrived at facility, wait to see provider was too long	na	na	na	na	na	na		na	na	3%	2%	na	na
Facility doesn’t provide method because it’s religiously affiliated	na	na	na	na	na	na		na	0%	na	na	1%	1%
Didn’t like treatment/treated unfairly by staff at facility	na	na	na	na	na	na		4%	8%	4%	7%	6%	4%
**Confidentiality/Privacy**													
Didn’t trust giving personal information to medical personnel^c^	0%	1%	na	na	1%	1%		0%	0%	na	na	0%	3%
Didn’t want the insurance holder to see the visit on insurance statement	na	2%	na	na	6%	2%		na	na	na	na	na	na
Didn’t want partner/family to know they were trying to get a birth control method	na	na	na	na	na	na		8%	7%	na	na	3%	6%
Had concerns about privacy/confidentiality	na	na	na	na	na	na		na	na	na	3%	na	na
**Personal or other**													
Didn’t feel safe getting health care services	na	2%	na	na	1%	1%		na	3%	na	na	1%	0%
Haven’t decided on a birth control method	na	na	na	na	na	na		na	na	16%	na	na	na
Some other reason	1%	3%	4%	2%	2%	3%		9%	17%	11%	14%	18%	17%
**Didn’t answer**	2%	3%	1%	4%	2%	3%		0%	4%	0%	0%	0%	1%

Notes: Samples are weighted to reflect females aged 18–44 (Surveys of Women) or female family planning clinic patients aged 18–44 (Family Planning Clinic Patient Surveys) within each state; samples include respondents who reported not receiving wanted health care in the past 12 months and who reported at least one reason for not receiving care. Samples include respondents who reported delay or trouble getting birth control in the last 12 months and who reported at least one reason for delay or trouble getting birth control. The presence of ’na’ indicates the response option not provided in the survey.

^a^Reasons for not receiving wanted health care in the past 12 months are not mutually exclusive; respondents could indicate as many reasons as desired and thus do not sum to 100%. ^a^Reasons delayed or had trouble getting desired birth control in last 12 months are not mutually exclusive; respondents could indicate as many reasons as desired and thus do not not sum to 100%.

^b^The response options differed slightly across surveys. In the Iowa clinic-based and statewide surveys, this response was ’I did not have transportation or a ride to the clinic/pharmacy’. The response in all other surveys was ’It was too hard to get to (no transportation or child-care, couldn’t take time off work)’.

^c^The response options differed slightly across surveys. In the Arizona clinic-based survey, this response was ’I didn’t trust giving out my personal information to medical staff’ as a reason for not receiving wanted healthcare in the past 12 months. The response in all other surveys was ’I didn’t trust giving out my personal information to medical personnel’.

### Logistic regression models

Among all populations except Wisconsin family planning clinic patients, those with no health insurance had significantly greater crude odds of being delayed or having trouble getting desired birth control in the past 12 months than those with health insurance (Table 4). In models adjusting for age, race/ethnicity, sexuality, nativity, marital status, employment status, education and household income, all significant bivariate associations held. Adjusting for other characteristics only slightly attenuated the odds for most populations; for Iowa the odds increased slightly.

**Table 4:** Associations between having no health insurance and being delayed or had trouble getting desired birth control in the last 12 months among women aged 18–44 in Surveys of Women and Family Planning Clinic Patient Surveys in Arizona, Iowa, Wisconsin, 2018–2020.

	Reported delays or trouble in getting desired birth control in the last 12 months							
	Family Planning Clinic Patients				Survey of Women			
Uninsured (ref: insured)	OR	95% C.I.	aOR	95% C.I.	OR	95% C.I.	aOR	95% C.I.
**Arizona**	2.0	(1.2 - 3.1)	2.0	(1.3 - 3.1)	2.8	(1.5 - 4.9)	2.2	(1.2 - 4.1)
**Iowa**	3.4	(2.0 - 5.9)	3.6	(2.0 - 6.2)	3.8	(1.9 - 7.4)	3.4	(1.4 - 8.2)
**Wisconsin**	1.7	(0.9 - 3.0)	1.4	(0.8 - 2.6)	4.8	(2.5 - 9.3)	4.3	(2.2 - 8.2)

Notes: Samples are weighted to reflect females aged 18–44 (Surveys of Women) or female family planning clinic patients aged 18–44 (Family Planning Clinic Patient Surveys) within each state. aORs come from multivariable logistic regression models of weighted data controlling for the following demographic characteristics: employed (y/n), income % FPL, education, age, race and ethnicity, sexual orientation, nativity, and relationship status.

Notes: The n for analytic samples for each set of models is: Arizona Family Planning Clinic Patients n = 1,379, Arizona Survey of Women n = 1,758; Iowa Family Planning Clinic Patients n = 1,073; Iowa Survey of Women n = 2,171; Wisconsin Family Planning Clinic Patients n = 1,846, Wisconsin Survey of Women n = 1,812.

## Discussion

We found that in Wisconsin, Iowa, and Arizona each, among both all women aged 18–44 and family planning clinic patients specifically, a high proportion (80% and greater) had used a method of birth control in the past three months. These findings are higher than similar outcomes reported in state-level data, which indicate that 68–72% of women of a wider reproductive age range (18–49) in Iowa, Wisconsin and Arizona were using a contraceptive method in 2019 [24]. Similarly, the majorities of all study populations reported having a personal healthcare provider; although at least one quarter of family planning patients in each state indicated not having one. These findings can be placed in the context of national-level evidence from publicly funded family planning patients indicating that, for most, their family planning provider is their only source of medical care. [25]; this highlights the critical role that publicly funded family planning sites play as a key healthcare provider embedded within the broader health care system. Among those patients and individuals who had received recent contraceptive care, about half or more of each study population rated their provider as “excellent” on four measures of patient centered care. These findings are in line with national-level evidence on the prevalence of this experience: in 2017–2019, 51% of women who had received recent contraceptive care were considered to have received person-centered contraceptive care according to the same metric [26]. Still, given the importance of a person-centered approach to the delivery of high-quality contraceptive care, the varying percentages of patients and individuals in our study reporting having received this care, and the small but not negligible numbers of study participants who reported provider-related mistrust or mistreatment, there is more room for improvement for providers to take their patients’ contraceptive needs and preferences seriously, provide adequate information for patients to make fully-informed decisions about contraception, and respecting these decisions, thus better meeting their patients’ needs.

The majority of all populations also had some form of health insurance, though these proportions vary by state and population. Notably, although Wisconsin is the only study state that did not expand Medicaid eligibility above poverty under the ACA, the percent of Wisconsin family planning clinics patients without health insurance appears to be similar to that of Iowa and is about 1/4 that of Arizona family planning clinic patients, among whom 26% had no health insurance. Wisconsin also has expanded Medicaid eligibility in the specific context of family planning care, which may be contributing to the high levels of Medicaid coverage among Wisconsin family planning patients documented in our study. These results are also consistent with a study that found that from 2014–2018, Wisconsin had a lower percent of adults that were uninsured than both expansion and non-expansion states [27]. This may, in part, be due to its alternative approach to health insurance coverage, specifically that the state covers adults up to 100% of the federal poverty level under Medicaid [28, 29].

Still, across the study populations, at least 20% reported not getting desired health care, and between 10% (Iowa and Wisconsin aged 18–44) and 19% (Arizona family planning clinic patients) had trouble or delays in obtaining their desired method of birth control in the past year, indicating that a significant proportion of women in all study populations are not fully getting their sexual and reproductive health care needs met. These levels of not being able to access desired health care and trouble in accessing contraception among reproductive-aged women are comparable to those documented among a similar population in Ohio during the same timeframe [15]. We found that cost is a primary barrier to healthcare services broadly and desired contraception specifically. Across study populations, those who had trouble or were delayed in obtaining their desired contraceptive method in the past year, the preponderance reported lack of health insurance and issues associated with the cost of care (e.g. not being able to afford it and inadequate health insurance), as well as problems arranging such care that often involve monetary costs to overcome (e.g. lack of transportation and child care) as reasons such trouble and delays. Our statistical model was consistent with these outcomes. After controlling for other characteristics often associated with contraceptive access and use, not having health insurance remained strongly associated with having been delayed or having trouble obtaining a desired contraceptive in the last year, except among Wisconsin family planning clinic patients.

A unique strength of this study is that we describe measures of health care access and use collected from three US states that experienced changes in healthcare and family planning funding policy across two key populations: all adult women aged 18–44 and women of the same age who sought family planning services at a publicly funded healthcare facility. Importantly, the latter population represents individuals who were able to access sexual and reproductive health care, so barriers to care among this group can be expected to be greater for those not able to access this care, who are not represented in our patient samples. We advise readers to exercise extreme caution in directly comparing the full population of adult women aged 18–44 and adult women family planning clinics patients within and across states for several reasons. First, survey data were collected using different sampling and weighting techniques and distributions and associations were estimated separately. Second, these populations are not mutually exclusive, and given the sampling strategies, undoubtedly people who use publicly funded family planning facilities are represented among the statewide survey population data. Third, the fact that both the implementation of the Title X Final Rule in 2019 and the declaration of the COVID-19 global pandemic as a public health emergency in 2020 occurred at varying stages during each survey fielding period further limits the ability to make direct comparisons between datasets. These study findings, especially those most malleable to differences across state settings such as health insurance coverage. must be considered in the context of these noted broader events. Still, we present results from each survey side by side to provide data points and context for state level advocate and policymakers who need to understand where gaps in access can still be addressed.

These data serve as a baseline to monitor access and use of reproductive health services in Arizona, Wisconsin, Iowa in the wake of drastic family planning funding shifts that changed the availability and capacity of the family planning service infrastructure across the country. While the majorities of our study populations report access to healthcare services of some kind, including contraceptive care, there are significant proportions of women for whom the cost of obtaining care remains a barrier to timely, desired services. During the period of data collection for this study across the states (2018–2021), political and legislative support for sexual and reproductive health care and rights varied across the states, with more hostile climates in Arizona and Iowa, and a mixture of hostility and support between the legislative and executive branches in Wisconsin [3]. With Arizona becoming more supportive following the 2022 elections and the US Supreme Court overturning *Roe v*. *Wade* in 2022, continuing to monitor the sexual and reproductive health care access metrics presented in this study will help to shed light on the potential broader impact of these political shifts.

## Supporting information

S1 Appendix Comparison of Surveys of Women (SoW) estimates to American Community Survey (ACS) 1-year estimates and their respective reference. (PDF)
