# Supplementary material for: Primary and reproductive healthcare access and use among reproductive aged women and female family planning patients in 3 states
Source: PLoS One. 2023 May 24;18(5):e0285825. doi: 10.1371/journal.pone.0285825 (PMC10208491; doi:10.1371/journal.pone.0285825)
Supplement: S1 Appendix — (PDF) [file pone.0285825.s001.pdf]

S1 Appendix. Comparison of Surveys of Women (SoW) estimates to American Community Survey (ACS) 1- year estimates and their respective reference year

	Arizona		Iowa		Wisconsin	
	SoW (2019)	ACS (2019)	SoW (2018)	ACS (2018)	SoW (2019)	ACS (2019)
Age						
18-24	26%	27%	27%	29%	24%	27%
25-29	20%	20%	18%	18%	19%	19%
30-34	19%	18%	19%	17%	19%	18%
35-39	19%	18%	18%	19%	20%	19%
40-44	17%	17%	18%	16%	18%	17%
Foreign born[a]	17%	17%	8%	7%	8%	6%
Married[b]	43%	43%	48%	49%	47%	45%
Education						
Less than high school	6%	11%	2%	7%	3%	6%
High school graduate, GED or alternative	18%	24%	14%	21%	15%	22%
Some college or Associate degree	49%	37%	49%	39%	45%	37%
College graduate or more	27%	28%	34%	32%	36%	35%
Income less than 100% of the federal poverty level	17%	16%	14%	17%	14%	15%
No insurance[c]	12%	19%	5%	10%	6%	12%

Notes: 2018 and 2019 1-year ACS estimates were obtained from the the U.S. Census Bureau's data tabling interface (https://data.census.gov/); some additional processing of ACS data was performed to limit data to women of reproductive age. Data represent women 18-44 unless otherwise specified.

[a]SoW includes women 18-44, ACS includes women 18 years of age and older

[b]SoW includes women 18-44, ACS includes women 20-44

[c]SoW includes women 18-44, ACS includes women 19-44
